# Structures of distant diphtheria toxin homologs reveal functional determinants of an evolutionarily conserved toxin scaffold

**DOI:** 10.1038/s42003-022-03333-9

**Published:** 2022-04-19

**Authors:** Seiji N. Sugiman-Marangos, Shivneet K. Gill, Michael J. Mansfield, Kathleen E. Orrell, Andrew C. Doxey, Roman A. Melnyk

**Affiliations:** 1 Molecular Medicine Program, The Hospital for Sick Children Research Institute, 686 Bay Street, Toronto, M5G 0A4 ON Canada; 2 Department of Biochemistry, University of Toronto, Toronto, M5S1A8 ON Canada; 3 Genomics and Regulatory Systems Unit, Okinawa Institute of Science and Technology Graduate University, Onna, Okinawa Japan; 4 Deparment of Biology, University of Waterloo, Waterloo, ON Canada

**Keywords:** Pathogens, X-ray crystallography, Transferases, Molecular evolution

## Abstract

Diphtheria toxin (DT) is the archetype for bacterial exotoxins implicated in human diseases and has played a central role in defining the field of toxinology since its discovery in 1888. Despite being one of the most extensively characterized bacterial toxins, the origins and evolutionary adaptation of DT to human hosts remain unknown. Here, we determined the first high-resolution structures of DT homologs outside of the *Corynebacterium* genus. DT homologs from *Streptomyces albireticuli* (17% identity to DT) and *Seinonella peptonophila* (20% identity to DT), despite showing no toxicity toward human cells, display significant structural similarities to DT sharing both the overall Y-shaped architecture of DT as well as the individual folds of each domain. Through a systematic investigation of individual domains, we show that the functional determinants of host range extend beyond an inability to bind cellular receptors; major differences in pH-induced pore-formation and cytosolic release further dictate the delivery of toxic catalytic moieties into cells, thus providing multiple mechanisms for a conserved structural fold to adapt to different hosts. Our work provides structural insights into the expanding DT family of toxins, and highlights key transitions required for host adaptation.

## Abstract (web summary)

Toxic proteins are responsible for the disease symptoms accompanying bacterial infections. Sugiman-Marangos et al. report crystal structures of two diphtheria toxin homologs, therefore providing structural insights into this family of toxins and the evolutionary adaptation of these toxins to human hosts.

## Introduction

**Fig. 1:**
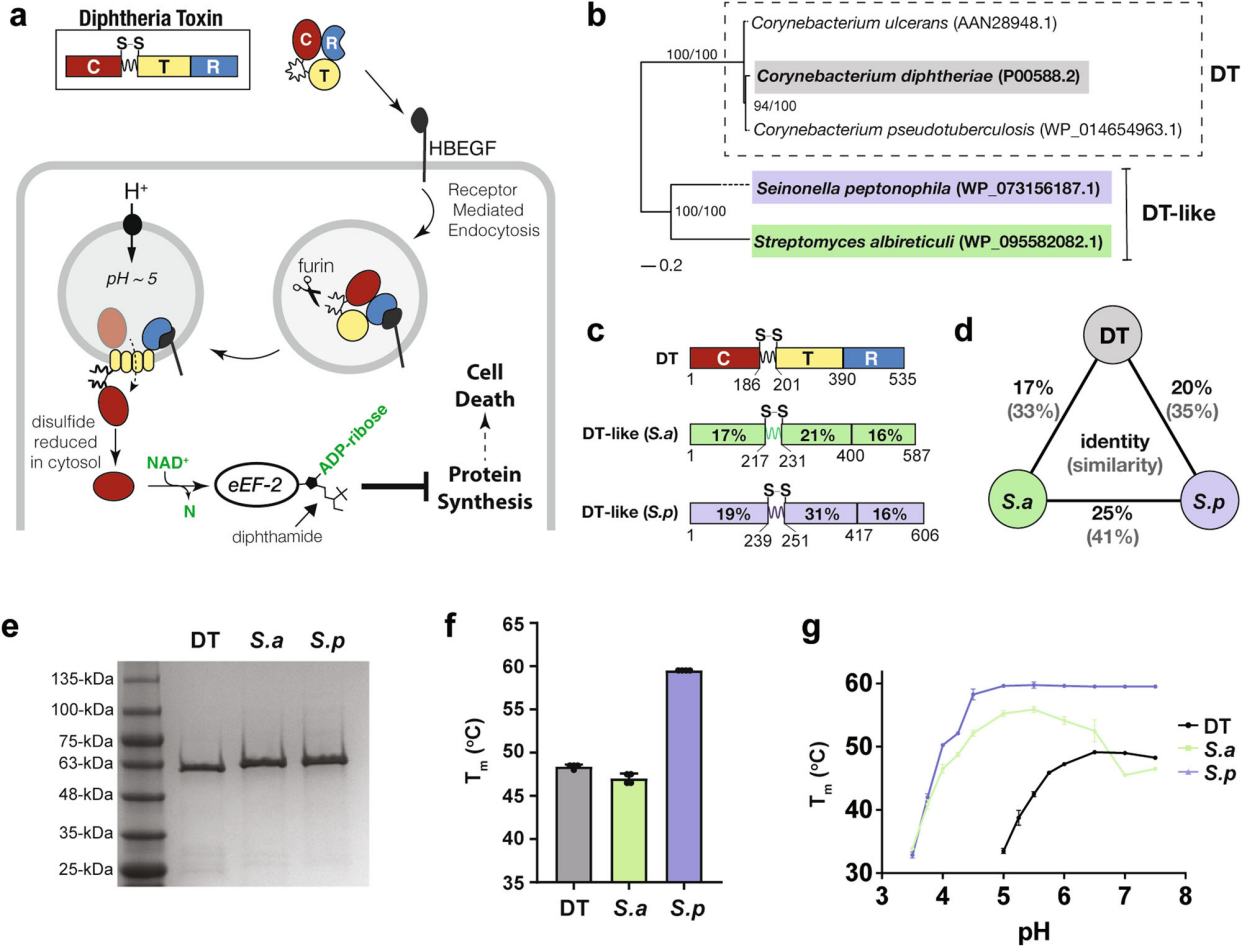
Distant homologs of diphtheria toxin retain the domain architecture of DT. **a** DT intoxication pathway. Briefly, the C-terminal **R**eceptor binding domain of DT (blue) triggers receptor mediated endocytosis upon binding its receptor Heparin Binding EGF Like Growth Factor (HBEGF). In the endosome, a furin-like protease cleaves between the **C**ytotoxic domain (red) and the **T**ranslocation domain (yellow) which remain tethered by an intramolecular disulfide bond. Acidification due to endosomal maturation results in conformational changes in the T domain which then inserts into the membrane, facilitating the escape of the C domain into the cytosol. The reducing environment of the cytosol reduces the disulfide bond, freeing the C domain to ADP-ribosylate its target eEF-2. ADP-ribosylation of the diphthamide residue on eEF-2 inactivates it, leading to protein synthesis inhibition and cell death. **b** Phylogenetic tree. DT and DT-like proteins from *S. albireticuli* (DT-like *S.a*) and *S. peptonophila* (DT-like *S.p*) are highlighted in gray, green and blue, respectively. Clade support values correspond to maximum likelihood bootstrap values from RAxML and posterior probability percentages from MrBayes. **c** Domain architecture and residue boundaries of DT, *S.a*, and *S.p*. **d** Three-way sequence identity and similarity (brackets) between DT, *S.a*, and *S.p*. **e** Purity of DT and DT-like proteins by sodium dodecyl sulfate polyacrylamide gel electrophoresis. **f** Melting temperature of DT, *S.a* and *S.p* at pH 7.5 as determined by differential scanning fluorimetry (mean ± SD, *n* = 4); *n* = 3 biological replicates. **g** pH stability of DT, *S.a* and *S.p* across a range of pHs measured using differential scanning fluorimetry (mean ± SD, *n* = 4); *n* = 3 biological replicates.

The disease symptoms that accompany many bacterial infections are caused by the actions of pathogen-derived proteins known as exotoxins. Since the discovery of the first bacterial exotoxin diphtheria toxin (DT) in 1888, as the cause of major symptoms of diphtheria, the field of toxinology has made considerable progress in understanding the structures and functions of these remarkably toxic proteins. The most potent toxins, such as botulinum toxin, tetanus toxin and diphtheria toxin, despite having no discernable sequence similarities, all shared a common architecture of three functional units: a cytotoxic enzymatic domain (C), a translocation domain (T), and a receptor binding domain (R). Nearly a century of research on the structure, function, and mechanisms of action of DT has uncovered insights into how this multifunctional protein binds, penetrates and intoxicates host cells to ultimately cause disease (Fig. 1a)^1^. Our knowledge of the evolutionary origins and molecular ancestry of DT, by contrast, remains poor due in large part to the lack of characterized relatives of DT. Traditional identification of novel toxins involves clinical isolates from bacterial infections of humans or livestock. As a result, the field of toxinology has been human-centric, complicating investigations of toxin evolution since ancestrally related proteins need not be related to pathogenesis in human hosts. Advances in rapid genome sequencing technologies have provided access to vast quantities of data which has led to the discovery of a variety of interesting bacterial toxins that might not otherwise have been studied^2^. This includes the identification of new members of the botulinum neurotoxin superfamily^3^, anthrax toxin family^4^, large clostridial toxin family^5^ and our recent identification of DT homologs outside of *Corynebacterium*^6^.

The recent discovery of DT homologs has provided a unique opportunity to study the origins of DT and potentially understand the determinants of its toxicity toward humans. Until this discovery, the only known homologs (from *C. ulcerans* and *C. pseudotuberculosis*) were too closely related (shared ≥94% sequence identity) to provide meaningful insights into the factors contributing to DT’s host specificity. Studying these distant homologs, which are found in organisms that have not been implicated as human pathogens, provides an opportunity to better understand the determinants of DT’s mechanism of action and toxicity in humans.

In this study, we structurally and functionally characterized two of the recently discovered DT-like homologs: one from *Streptomyces albireticuli*, a potential plant growth-promoting bacterium isolated from rhizosphere soil;^7^ and the other from *Seinonella peptonophila*, also isolated from soil (Fig. 1b). These DT-like proteins from non-human associated organisms appear to contain all the necessary domains to constitute legitimate exotoxins (Fig. 1c) and were chosen for investigation as they are the most divergent homologs from DT (17 and 20% sequence identity to DT, respectively), and, moreover, are nearly as dissimilar to one another (25% sequence identity) as they are to DT (Fig. 1d). Thus, these two DT-like proteins offer the greatest opportunity to elucidate the core determinants of toxicity shared by the DT family of toxins, and also identify the differences that underly the nature of host specificity. Through the elucidation of the first crystal structures of DT-like proteins and an analysis of their functions, we uncovered molecular features that define this family of toxins and dictate their host specialization.

## Results

### Production and biophysical characterization of two remote homologs of DT

To characterize the DT-like proteins from *Streptomyces albireticuli* and *Seinonella peptonophila*, their gene sequences (WP_095582082.1 and WP_073156187.1 respectively) were synthesized, incorporating codons that were optimized for expression in *E. coli*. Assembled sequences were each designed to also encode an amino-terminal cleavable Hexa-His-SUMO affinity tag to facilitate with purification that was removed using SUMO protease to liberate tag-less versions of both DT-like proteins (Fig. 1e).

Since we had no *a* priori knowledge of the specific environmental niche that the bacteria that produce these proteins naturally inhabit, we measured the folding and thermal stability of each purified DT homolog under different conditions. Initially, we evaluated the thermal stability of each DT-like protein, under neutral pH conditions (pH 7.5) using differential scanning fluorimetry (DSF) (Fig. 1f). Whereas the homolog from *S. albireticuli* (*S.a*) displays a T_m_ comparable to DT (46.5 °C cf. 48.25 °C), the homolog from *S. peptonophila* (*S.p*) is significantly more thermostable (*T*_m_ = 59.5 °C). To investigate this further, we performed the DSF analysis over a range of pH values (7.5–3.0). Notable differences in both the pH optimum range and pH dependence were observed: While the *T*_m_ of DT remains stable between pH 7.5 and pH 6 and decreases steadily as pH drops below 6, both homologs undergo a pH dependent drop in stability at a much lower pH (i.e., below ~pH 4.5). Interestingly, *S.a* appears to have a pH optimal range centered around pH 5.2, whereas *S.p* displays a constant Tm across a broad three pH unit range above pH 4.5 (Fig. 1g). These results show that *S.a* and *S.p* are generally more thermostable than DT at a given pH, and further require more acidic conditions than DT to unfold.

### High-resolution structural determination

Crystals of affinity purified tag-less DT-like proteins were grown by hanging-drop vapor diffusion and diffraction data was collected on the AMX beamline at NSLSII. Neither homolog could be solved by molecular replacement with the structure of DT, and therefore both required experimental phases derived from single-wavelength anomalous dispersion (SAD) datasets to be solved. Experimental phase for the DT-like protein from *S. albireticuli* was obtained from a 2.7 Å SAD dataset using selenomethionine (SeMet) derivatized protein crystals. As crystals of the DT homolog from *S. peptonophila* were grown in a condition containing potassium iodide, a 2.2 Å dataset was collected with native protein crystals at a wavelength of 2.0 Å to maximize the anomalous signal from iodide. The structure was subsequently solved using iodide-SAD phasing. A complete list of diffraction data and model refinement statistics for both structures can be found in Table 1.

**Table 1:** Data collection, phasing, and refinement statistics.

	Albireti toxin (AT)	Peptono toxin (PT)
*Data collection*		
Space group	P2_1_2_1_2	P2_1_2_1_2_1_
Cell dimensions		
* a*, *b*, *c* (Å)	106.95, 137.08, 203.51	77.65, 82.46, 91.82
*α*, *β*, *γ* (°)	90, 90, 90	90, 90, 90
Resolution (Å)	60.8–2.69 (2.696–2.687)^a^	48.14–2.19 (2.192–2.185)
*R*_merge_	18.9 (143.5)	11.8 (58.7)
*R*_pim_	5.2 (38.3)	3.5 (35.3)
*I*/σ*I*	10.9 (2.1)	16.4 (1.9)
Completeness (%)	99.9 (100.0)	92.7 (54.0)
Redundancy	13.9 (14.8)	11.5 (3.3)
CC_1/2_	99.8 (83.3)	99.6 (66.3)
Wilson *B*-factor	49.43	28.59
*Refinement*		
Resolution (Å)	2.69	2.19
No. of reflections	83,614 (8257)	28,814 (1832)
*R*_work_/*R*_free_	20.24/24.75	20.15/22.50
No. of atoms	18,286	5080
Protein	17,703	4662
Ligand/ion	156	26
Water	511	392
*B*-factors	52.13	33.38
Protein	52.48	33.08
Ligand/ion	56.56	61.03
Water	39.61	35.15
R.m.s. deviations		
Bond lengths (Å)	0.006	0.002
Bond angles (°)	0.79	0.50

^a^Values in parentheses are for the highest-resolution shell.

### Remote DT-like proteins display structural homology to DT

**Fig. 2:**
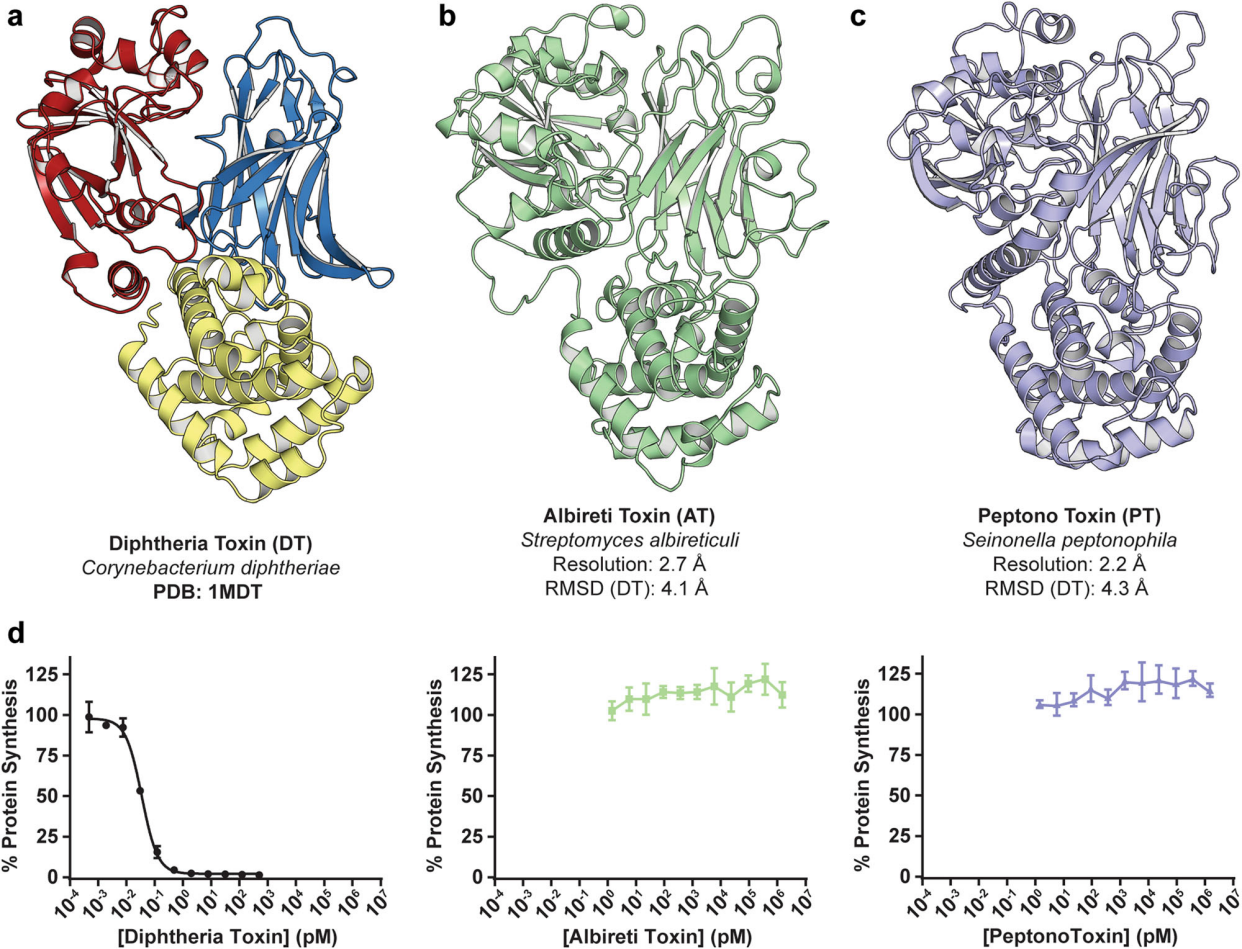
Structures and functions of remote diphtheria toxin homologs. **a** Crystal structure of DT. **b** Crystal structure of AT solved by SAD-phasing and refined to *R* and *R*_free_ values of 20.2% and 24.8% (PDB: 7RI3). The four monomers in the ASU align to each other with RMSDs of <1.0 Å. A complete model was built in each of the four chains, except for 4–5 residues from the N-terminus and 0–10 residues from the loop joining the catalytic and translocation domains. Shown is the most complete of the four chains, **c** crystal structure of Peptono Toxin solved by SAD-phasing and refined to *R* and *R*_free_ values of 20.2 and 22.5% (PDB: 7RB4). A complete model was built for the monomer in the ASU, apart from the first three N-terminal residues. **d** Dose titrations of DT, AT, and PT on DT-sensitive Vero cells (mean ± SD, *n* = 4); *n* = 3 biological replicates.

Both DT-like proteins, despite their low identity to DT and to each other (Fig. 1d and Supplemental Fig. 1), were found to adopt the characteristic three domain Y-shaped architecture of DT (Fig. 2a–c). Moreover, each of the individual domains preserve their respective folds: the central split β-sheet motif in the catalytic domain characteristic of the ADP-ribosyl transferase (ADPRT) superfamily; the three-layered α-helical translocation domain; and, the Ig-like jelly-roll fold in the receptor-binding domain. With respect to insertions and deletions, the T-domain appears to be the most structurally conserved of the three domains, while both the C and R-domains contain several additional secondary-structure features not present in DT. Structural alignments performed with the DALI server^8^ calculate the overall RMSD of the structures to DT as 4.1 Å (*S. albireticuli*—468/583 residues) and 4.3 Å (*S. peptonophila*—457/603 residues). Additionally, both DT homologs maintain the pinched disulfide loop at the junction between the C- and T-domains. Given these structural similarities, and for purposes of clarity and simplicity, we refer to these two DT homologs hereafter as Albireti Toxin (AT) and Peptono Toxin (PT).

### AT and PT holotoxins are non-toxic to DT-sensitive Vero cells

The striking structural conservation despite low sequence conservation of AT and PT with DT prompted us to explore their potential to function as toxins. AT and PT were added to Vero cells and incubated overnight; protein synthesis was measured using a nanoluciferase (nLucP) based assay^9^ following an overnight (18 h) incubation. Vero cells express high levels of the DT-receptor^10^, HBEGF, and are therefore particularly sensitive to DT. Under these conditions DT enters cells and inhibits protein synthesis with an EC_50_ of ~0.15 pM (Fig. 2d). By striking contrast, we found that AT and PT do not display any measurable toxicity on Vero cells even at µM concentrations (Fig. 2d). The observation that these DT homologs exhibited no cellular toxicity on Vero cells while having remarkable structural homology to DT, provided an unprecedented opportunity to elucidate the determinants of toxicity for DT. To this end, we carried out an in depth, domain-by-domain, functional/structural comparison with the goal of better understanding DT’s potency in human hosts.

### AT_R_ and PT_R_ share the same structural fold as DT_R_ but do not bind hHBEGF

**Fig. 3:**
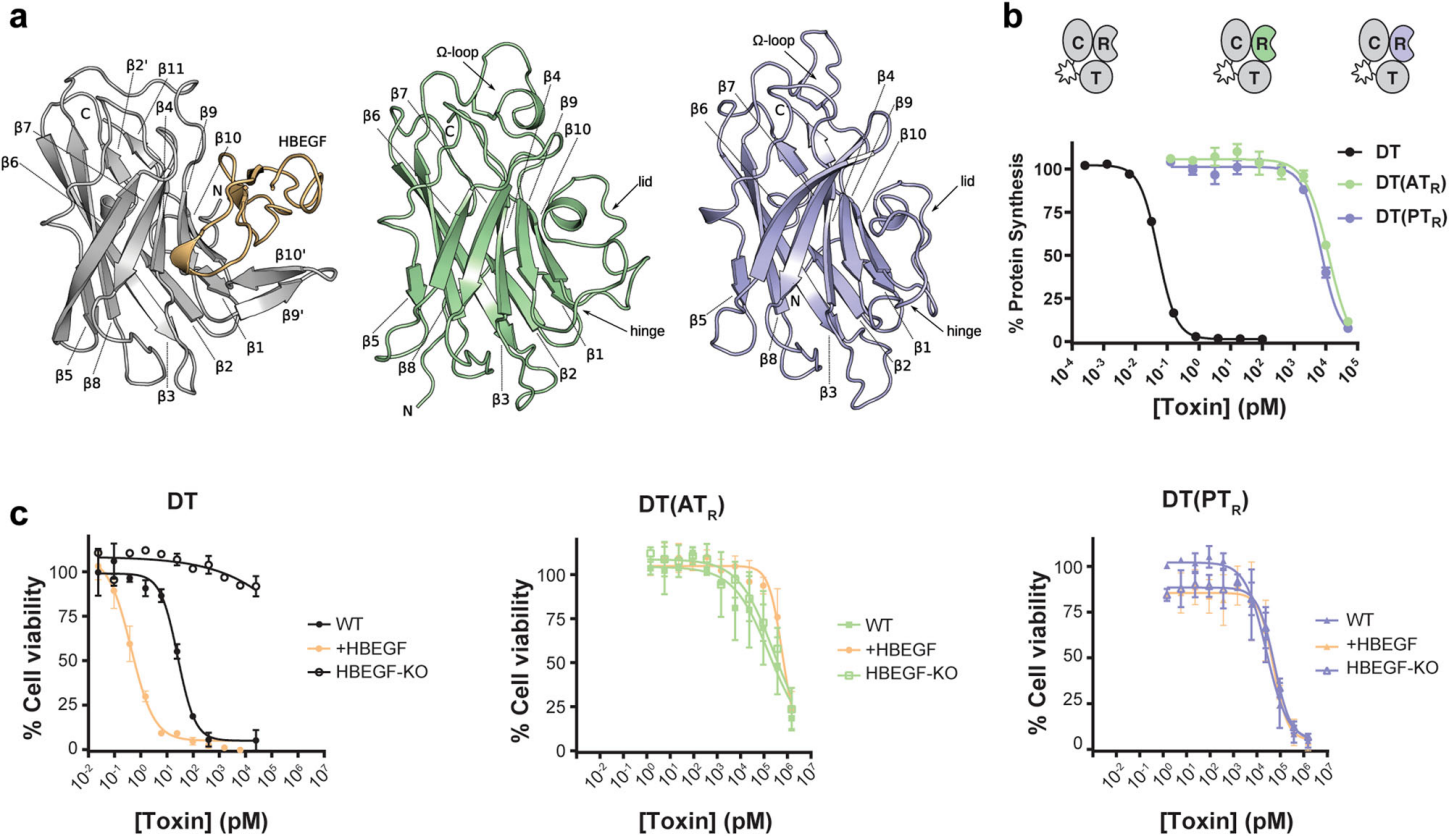
R-domains of AT and PT do not bind HBEGF. **a** R-domain structural comparison highlighting conserved β-sandwich motif. The structure of DT_R_ in complex with HBEGF (yellow) was generated from PDB entry 1XDT. The Ω-loop between β8 and β9, and the lid structure between β9 and β10 are labeled. **b** Dose titration of DT and DT R-domain chimeras DT(AT_R_), and DT(PT_R_) on Vero cells (mean ± SD, *n* = 3); *n* = 3 biological replicates. **c** Dose titration of DT, DT(AT_R_), and DT(PT_R_) on WT, HBEGF_KO_ and +HBEGF cells (mean ± SD, *n* = 2); *n* = 3 biological replicates. DT sensitivity (EC_50_) tracked with expression of HBEGF (WT—26 pM, HBEGF_KO_-N/A, + HBEGF—0.43 pM). Sensitivity to DT(AT_R_) and DT(PT_R_) was unaffected by HBEGF expression (WT—169 and 25 nM, HBEGF_KO_—245 and 55 nM, +HBEGF—608 and 50 nM).

Of the three domains making up the holotoxin, the receptor-binding domains of AT and PT have the lowest sequence conservation with DT (16% and 13% sequence identity, respectively). We previously noted that as these regions are highly divergent in sequence and were not recognizable using existing DT_R_ domain models^6^, they likely play a key role in determining receptor/host specificity. Despite marginal sequence identity with DT_R_, the X-ray crystal structures reveal that AT_R_ and PT_R_ are in fact highly similar to DT_R_, maintaining the core β-sandwich motif (β1-2-3-8-5 and β6-7-9-10) present in DT_R_ (Fig. 3a). Overall, AT_R_ and PT_R_ appear to be less compact than DT_R_, each containing ~30 additional residues distributed among the loop regions joining secondary structure elements in the β-sandwich. Most of these residues are contained within two insertions present in both AT and PT: a 10 amino acid Ω-loop (7 amino acids in PT) and a 16 amino acid insertion in the loop joining β9 and β10. In DT_R_, β9 and β10 contribute a significant proportion of the HBEGF binding surface. In the bound state, the distal end of the DT_R_ β9-10 hairpin bends inward forming a concave surface like a grasping hand, breaking the continuity of the strands (Fig. 3a). In AT_R_ and PT_R_, the 16 amino acid insertion between β9 and β10 contributes to an ~25 amino acid “lid,” hinging at AT_G550_ and AT_N576_ (PT_P571_ and PT_D596_), that folds back over and occludes the presumed receptor binding surface (Fig. 3a and Supplemental Fig. 2). The apex of the loop consists of a helical-turn that lies precisely in the groove where HBEGF is bound in the DT/HBEGF co-crystal structure.

To determine whether AT_R_ and PT_R_ bind and use human HBEGF (hHBEGF) as a cellular receptor, we generated chimeric constructs with DT in which the R domain of DT was replaced with the R domain from AT or PT. In each DT chimera, DT_R_ was removed and replaced with the corresponding domain from the homolog, e.g., in DT(AT_R_), DT_1-377_ was fused to AT_400-587_. A full description of domain boundaries used in DT chimeras is outlined in Supplemental Fig. 3. Chimeric DT(AT_R_) and DT(PT_R_) displayed greatly attenuated toxicity toward Vero cells with EC_50_ values of 12 and 7 nM, respectively (Fig. 3b). To confirm that the chimeric constructs do not engage HBEGF, we engineered ARN8 cells (that have basal levels of HBEGF) to have either no HBEGF (HBEGF-KO), or high levels of HBEGF ( + HBEGF). Whereas DT sensitivity tracked with expression of HBEGF (Fig. 3c), neither DT(AT_R_) nor DT(PT_R_) were affected by knockout or overexpression of HBEGF, further demonstrating that AT and PT do not recognize HBEGF as a cellular receptor. In fact, levels of toxicity for DT(AT_R_) and DT(PT_R_) were indistinguishable for a DT construct devoid of a receptor binding moiety (DT_1-389_) (Supplemental Fig. 2b).

Attempts to unmask a cryptic HBEGF binding site through genetic deletion of the lid in AT_R_ proved unsuccessful (Supplemental Fig. 2c), further demonstrating a lack of receptor recognition, and suggesting that the lids are not responsible for the lack of binding to HBEGF. Taken together, these data demonstrate that AT and PT are unable to bind HBEGF or any other human cell-surface protein as a receptor to enter cells, thus playing a major role in the inability of the AT and PT holotoxins to intoxicate human cells.

### AT and PT catalytic domains are potent modifiers of eEF-2

**Fig. 4:**
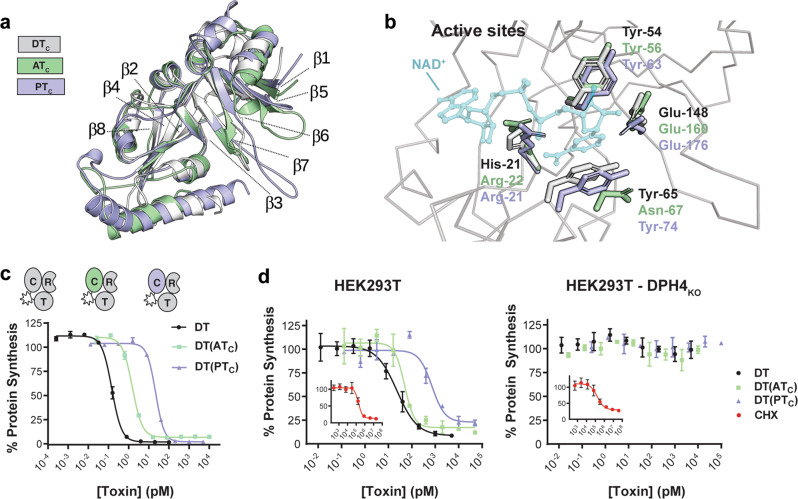
The catalytic moieties from AT and PT are cytotoxic. **a** Overlay of C-domains highlighting the conserved split β-sheet characteristic of ADPRT fold proteins. **b** Active site residue conservation. DT residues important for NAD^+^ binding and catalysis and corresponding residues in AT and PT are represented in stick form. NAD^+^ represented in partial transparency is modeled from PDB entry 1TOX for reference. **c** Dose titration of DT and DT C-domain chimeras DT(AT_C_), DT(PT_C_) on Vero cells (mean ± SD, *n* = 2); *n* = 3 biological replicates. **d** Dose titration of DT, DT(AT_C_), and DT(PT_C_) on HEK293T and HEK293T DPH4_KO_ cells (mean ± SD, *n* = 2); *n* = 3 biological replicates. Titration of protein synthesis inhibitor cycloheximide (CHX) is shown in red (inset).

We next evaluated the functions of the catalytic domains of AT and PT as structurally, the catalytic domains contain many of the hallmarks of a functional ADP-ribosyltransferase. Both AT_C_ and PT_C_ contain the characteristic split β-sheet (β1-5-6-7-3 and β2-4-8) at the core of the ADPRT fold (Fig. 4a) shared among other ADP-ribosylating toxins (Supplemental Fig. 4). Moreover, within the putative active sites, two key active site residues, Y54 and E148, are absolutely conserved and structurally equivalent in the two DT homologs (Fig. 4b). To evaluate whether the individual catalytic moieties of AT and PT had cytotoxic activity if introduced into cells, we generated domain-swapped chimeras between DT and AT_C_ or PT_C_ and determined their toxicity on DT-sensitive cells (Fig. 4c). Despite AT and PT holotoxins not displaying any toxicity on cells up to μM concentrations, DT(AT_C_) and DT(PT_C_) inhibited protein synthesis with EC_50_ values of 1.5 and 22 pM, respectively, as compared to wild-type DT with an EC_50_ value of 0.15 pM (Fig. 4c), indicating that AT_C_ and PT_C_ are in fact highly toxic if delivered into cells via DT_T_ and DT_R_.

Next, to determine whether the homologs induce cytotoxicity by targeting the same eEF-2 residue as DT (diphthamide), we examined a CRISPR knockout cell-line unable to synthesize diphthamide (HEK239T DPH4_KO_). Wild-type and DPH4 _KO_ cells were treated with increasing concentrations of DT, DT(AT_C_), DT(PT_C_) or a chemical inhibitor of protein synthesis (cycloheximide), and protein synthesis was assessed following overnight incubation (Fig. 4d). While both cell types were sensitive to cycloheximide, only the wild-type HEK293T cells were sensitive to DT and the catalytic domain chimeras. DPH4 _KO_ cells were unaffected by any of the toxins. The observed strict dependence on diphthamide biosynthesis demonstrates that AT_C_ and PT_C_ induce profound cytotoxicity through a DT_C_-like mechanism if introduced into cells.

### AT and PT lack the canonical protease activation site of DT

**Fig. 5:**
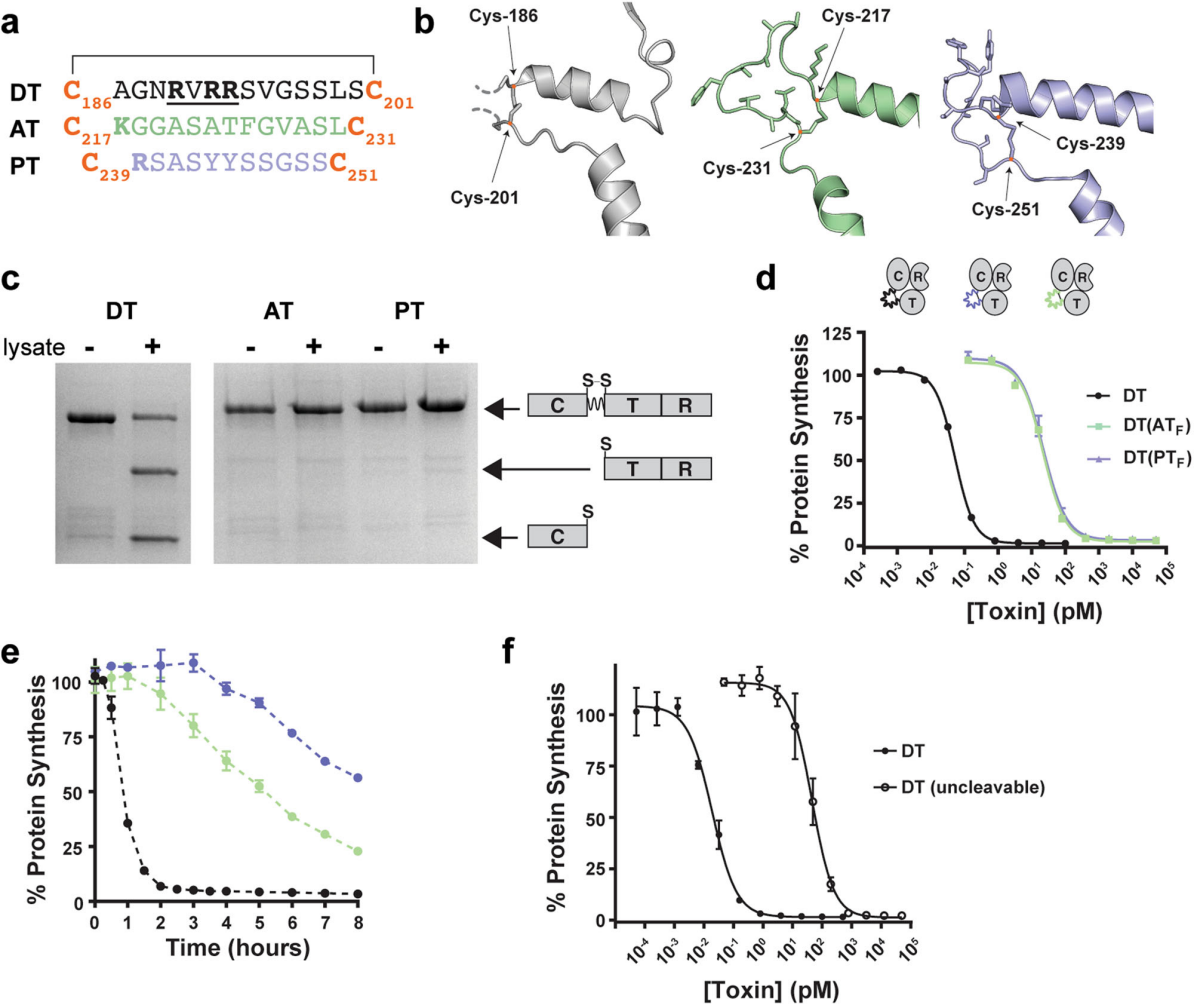
AT and PT are not efficiently processed. **a** Primary amino acid sequences of loops linking C and T domains in DT, AT, and PT. **b** Structures of processing sites in DT, AT, and PT in gray, green, and purple, respectively. **c** SDS-PAGE analysis of holotoxin processing by Vero cell lysate. **d** Dose titration of DT and DT activation loop chimeras DT(AT_F_), DT(PT_F_) on Vero cells (mean ± SD, *n* = 2); *n* = 3 biological replicates. **e** Time course of protein synthesis inhibition in Vero cells treated with 10 nM of DT, DT(AT_F_), and DT(PT_F_) (mean ± SD, *n* = 2); *n* = 3 biological replicates. **f** Dose titration of DT and DT (uncleavable) on Vero cells (mean ± SD, *n* = 4); *n* = 3 biological replicates.

Of the seven homologs we identified previously^6^, AT and PT were the only two lacking the canonical ‘RXXR’ furin cleavage motif in the loop joining the putative catalytic fragments with the downstream putative translocation and receptor binding fragments (Fig. 5a). Of note, however, AT and PT contain both Cys residues (C217/C231 and C239/C251, respectively) that bound this region, and the crystal structures confirm formation of the inter-fragment disulfide bonds (Fig. 5b). To determine whether the intervening loop could still be recognized and cleaved by another cellular protease, DT, AT, and PT were incubated with total cell lysate from Vero cells. To evaluate whether cleavage occurred, toxins were separated by SDS-PAGE under reducing conditions. Whereas DT was processed into its two fragments following overnight incubation with lysate, neither of the homologs showed any significant processing (Fig. 5c). The functional consequences of not releasing the cytotoxic ADPRT cargo into cells after translocation on toxicity was assessed. The residues subtended by C186 and C201 from DT were replaced with the corresponding residues from AT and PT and their toxicity was measured after 48-hours. The DT(AT_F_) and DT(PT_F_) chimeras were both approximately two orders-of-magnitude less potent than DT (Fig. 5d). To further investigate the functional consequences of having an ostensibly non-cleavable site in place of the furin processing site in DT, Vero cells were incubated with 10 nM of DT, DT(AT_F_) or DT(PT_F_), a concentration which after overnight incubation results in complete cell death, and protein synthesis was evaluated at regular intervals for a total of 8 h. Whereas cells treated with DT experienced a rapid onset of protein synthesis inhibition 1 h after toxin administration, cells treated with DT(AT_F_) and DT(PT_F_) demonstrated slower kinetics of inhibition of protein synthesis (Fig. 5e), highlighting a potential role for the proteolytic processing event in accelerating the intoxication process. Consistent with these findings, a version of DT wherein a non-cleavable linker corresponding to the tobacco etch virus protease recognition site (QGNENYLFQGSSLS) was introduced into this site (uncleavable), had a similar shift to DT(AT_F_) and DT(PT_F_), confirming that AT and PT lack canonical protease activation sites, and this only marginally effects the toxicity of DT (Fig. 5f).

### AT and PT translocation domains require lower pH for membrane insertion

**Fig. 6:**
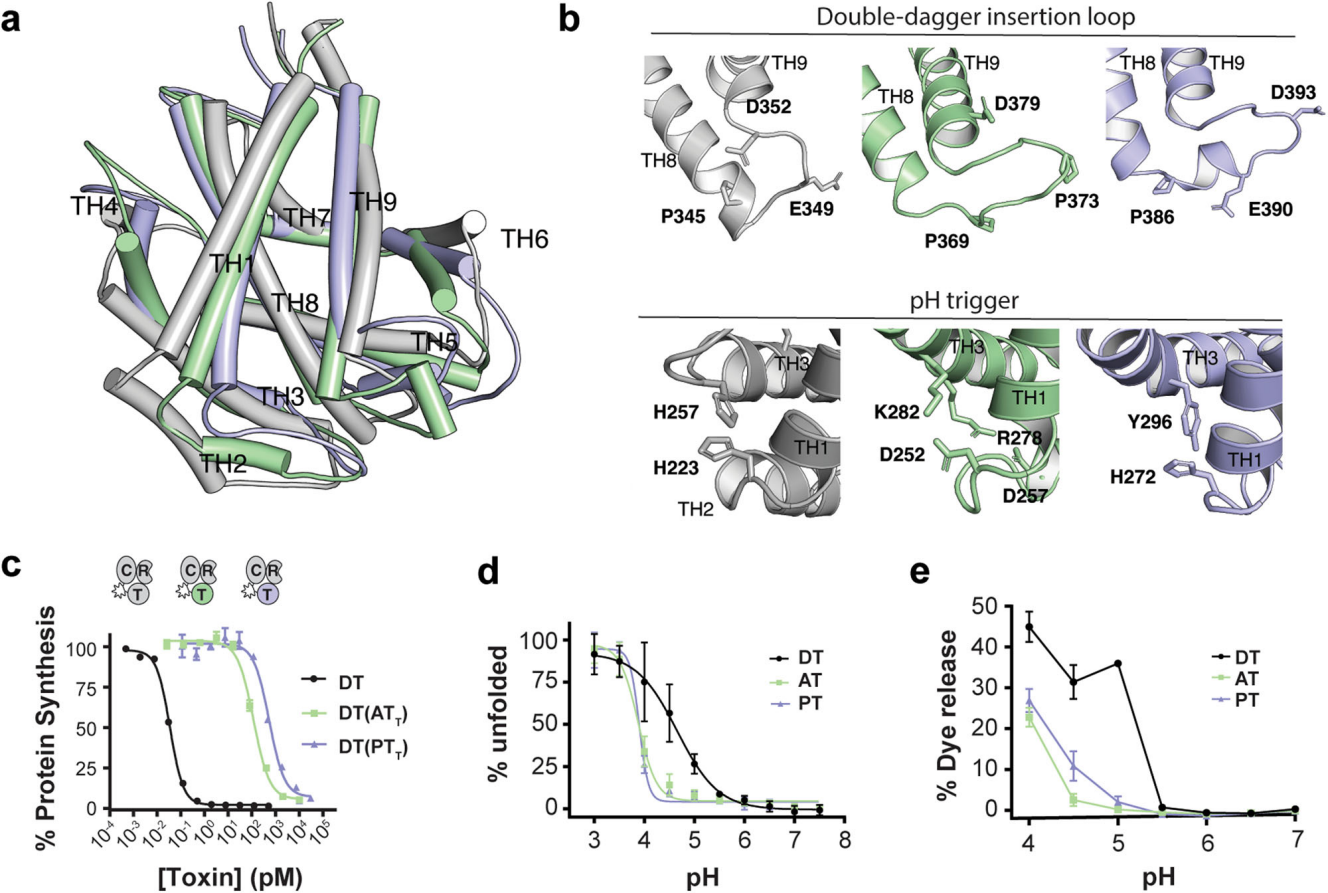
AT and PT have functional translocases with altered pH dependence. **a** Overlay of T-domain structures (AT: 20% sequence identity, RMSD 3.0 Å; PT: 31% sequence identity, RMSD 3.1 Å). **b** Residues of interest in the TH8/TH9 loop involved in initial membrane insertion of DT_T_ (top). AT and PT contain an equivalent to DT_P345_ (AT_P369_ and PT_P386_). The loop in AT contains a three-residue insertion and an additional Pro residue within the loop (AT_P373_), and only one negatively charged residue (AT_D379_). PT_P386_ causes a 90° kink in TH8, which continues for a full turn before terminating with a Glu residue (PT_E390_). Residues of interest at the junction of TH1, TH2, and TH3 believed to play an important role in early destabilization of DT_T_ (bottom). AT_T_ contains two salt-bridges (D252/R282, D257/K278) in lieu of His residues in this junction. **c** Toxicity of chimeric toxins on Vero cells. Dose titration of DT and DT T-domain chimeras DT(AT_T_), and DT(PT_T_) on Vero cells (mean ± SD, *n* = 2); *n* = 3 biological replicates. **d** pH dependent TNS analysis (mean ± SD, *n* = 3). **e** pH-dependent dye leakage from liposomes loaded with fluorophore quencher pair (HPTS/DPX). DT exhibits a spike of activity upon exposure to pH 5, while AT and PT are only active at pH 4, a full pH unit lower than DT (mean ± SD, *n* = 4); *n* = 3 biological replicates.

The T domain is arguably the most specialized domain of DT: the acidic environment of the early endosome induces partial unfolding and formation of a molten globule state followed by insertion of at least two marginally hydrophobic segments into the endosomal membrane to form a pore that catalyzes delivery of the toxic C domain into the cytosol^11–13^. Structurally, both AT_T_ and PT_T_ maintain the same three-layered helical architecture as DT_T_, only lacking structural equivalents to TH2 (PT) and TH4 (AT and PT) (Fig. 6a). Notably, the hydrophobic helices that make up the “double-dagger” motif (TH5-9)^13^ are particularly well conserved structurally, consistent with the disproportionate sequence conservation of the first half (TH1-4) vs the second half (TH5-9) of the T domains (AT—13% cf 21%, PT—25% cf 39%). The loop joining TH8 and TH9 is thought to initiate insertion into the endosomal membrane upon partial protonation of E349 and D352 during endosomal acidification^11–13^ (Fig. 6b). In addition to the two negatively charged residues, P345 is thought to play an important role in mediating membrane insertion of the T domain^14,15^. Both AT and PT contain an equivalent Pro residue at the C-terminus of TH8 that breaks the helix (Fig. 6b). The loop in AT contains only one negatively charged residue which sits within the first turn of TH9 rather than in the loop itself, while PT contains equivalents to both DT_E349_ and DT_D352_ (Fig. 6b).

To test whether the T-domains from AT and PT could function as protein translocases, as above we generated chimeric constructs with DT in which the T domain of DT was replaced with the T domain from AT or PT. DT(AT_T_) and DT(PT_T_) displayed toxicity to Vero cells with EC_50_ values of 117 and 535 pM, respectively (Fig. 6c), demonstrating that both AT_T_ and PT_T_ are functional translocases, albeit with significantly decreased apparent efficiency as compared with DT_T_. A potential clue to explain the reduced ability of AT_T_ and PT_T_ to act as translocases in Vero cells came from a closer look at the key residues implicated in the pH-trigger for DT (reviewed in ref. ^16^). Of the six His residues in DT_T_, two have been identified as playing particularly crucial roles in initial unfolding events: H223 and H257, located in the loops between TH1/2 and TH3/4, respectively (Fig. 6b). Protonation of H257 is thought to destabilize the first four helices of DT_T_^17^, exposing the hydrophobic helices (TH5-8) and thereby promoting their interaction with the membrane^18^. H223 has been proposed to act as a “safety latch” to prevent premature unfolding^19^. Remarkably, the T-domain of AT lacks His residues in the T domain entirely. Instead, this region (TH1-4) is stabilized by two salt-bridges between residues in TH1/TH3 and TH2/TH3 (Fig. 6b). The T domain of PT contains three His residues: H272, H276, and H364. Two of these residues are structurally equivalent with His residues in DT: PT_H272_ ↔ DT_H223_ and PT_H364_ ↔ DT_H322_. Rather than forming π-stacking interactions with a second His residue as in DT, PT_H272_ forms an edge-to-face π–π interaction with PT_Y296_ (Fig. 6b).

Based on differences in the pH-sensitive determinants for DT, and earlier biophysical characterization of the holotoxins (Fig. 1f, g), we posited that AT and PT may not be optimized to form functional translocases at early endosome pHs. To investigate this, we performed pH dependent 2-(p-toluidino) naphthalene6-sulfonic acid (TNS) analysis of the three proteins, which revealed that DT unfolds with a pKa of ~4.7, while the two remote homologs unfold with a pKa of ~3.9 (Fig. 6d). Next, we measured pH-dependent pore-formation using a liposome dye-release assay. Consistent with observed differences in residues implicated in the pH-sensor in DT, and increased pH stability (Figs. 1g and 6d), we found that AT and PT required a lower pH to unfold and form membrane-inserted pores (Fig. 6e) suggesting that these translocases are not optimized to form functional translocases in early endosomes.

## Discussion

We have presented here a structural and functional analysis of two distant homologs of DT, which has provided novel insights into the molecular evolution of the DT scaffold and the determinants of host specialization. This study was enabled by the recent discovery of the first distant DT-like relatives (i.e., all sharing <35% sequence identity with DT) from bacterial lineages outside of *Corynebacterium* with no known association with human hosts. Prior to this, the only known DT homologs were the highly related toxins from *C. ulcerans* and *C. pseudotuberculosis* (95% identity), which were highly toxic to human cells and offered little with respect to understanding the evolutionary history of DT. Moreover, the uniqueness of the structure of DT within the PDB made unraveling the molecular evolution of DT and the adaptations that enabled specialization towards human hosts difficult.

We found that the holotoxins from *S. albireticuli* and *S. peptonophila*, despite being virtually non-toxic to human cells, had strikingly similar structures to DT. Through a comprehensive domain-by-domain analysis, we showed that the factors that contribute to host cell toxicity are demonstrably localized within the B fragments of the toxins (binding, translocation and release), rather than the A fragments, which displayed comparable cytotoxicity if delivered into the Vero cell cytosol via the DT binding and delivery machinery. That the cytotoxic action of DT_C_ is functionally conserved in these distant homologs likely parallels the highly conserved nature of eEF2 across eukaryotes and is consistent with the observation that DT retains ADP ribosylation activity against yeast eEF-2^20^ and even archaeal eEF-2^21^. Importantly, these findings strongly suggest that AT and PT are “toxins” in a different context (i.e., host) where their respective B fragments are optimized to deliver the catalytic fragments into their respective hosts.

Within the catalytic fragment of DT, the residues that have been shown to be important for binding and coordinating NAD^+^ in the active site of DT are well characterized (H21, Y54, Y65, E148)^22–25^. These four residues are conserved within the active sites of other ADP ribosyltransferase enzymes including Exotoxin A, Cholix toxin and PARP. By contrast, in AT and PT, only Y54 and E148 are absolutely conserved: in both, an Arginine is seen in place of the H21 residue; and in AT an Asparagine is seen in place of Y65. Interestingly the pertussis toxin and cholera toxin ADPRT enzymes also have an Arginine at the position equivalent to DT_H21_ (Supplemental Fig. 3), indicating at least some functional equivalency in this position between Histidine and Arginine. Future studies on the substrate specificity and enzyme kinetics are needed to understand if these changes confer any additional unique properties to these enzymes.

As expected, the receptor binding domains of AT and PT proved to be important determinants of host specificity, with neither able to bind human HBEGF. Without any knowledge of the preferred host of these toxins, we are unable to distinguish at this time whether AT_R_ and PT_R_ bind HBEGF in their respective hosts, an HBEGF-like receptor, or an entirely different receptor. While the ‘lid’ structure is poorly conserved in terms of both sequence and structure (Supplemental Fig. 2a), the fact that it is present in both homologs raises several questions. Is the ‘lid’ merely a gate that can move out of the way to accommodate a receptor, or is the surface presented by the ‘lid’ responsible for mediating its receptor binding activity? Also, if this structural feature was present in the common ancestor of DT, AT, and PT, did the loss of this structural feature enable binding to receptors like HBEGF in non β-clade members of the family?

The highly attenuated proteolytic activation of AT and PT between A and B fragments and subsequent impact on toxicity (Fig. 5) highlights yet another evolutionary lever that can be pulled to tune host specificity. We show that total loss of protease activation results in a significant, albeit attenuated loss of cellular toxicity. Previously, two studies investigating the role of DT activation came to very different conclusions on the role of proteolytic release in DT. Tsuneoka et al. reported that furin-deficient LoVo cells were completely resistant to DT suggesting that proteolytic activation was absolutely essential for DT function^26^. By contrast, Gordon et al. showed only attenuated cytotoxicity of DT on furin-deficient cells^27^—similar to what was observed here. Differences in the kinetics of toxicity shown here (Fig. 5e) likely account for these differences, as was hypothesized previously by Gordon et al. This phenomenon has similarly been observed with another AB family toxin, TcdB from *Clostridium difficile*, where inactivation of the enzyme releasing domain results in a slight attenuation of toxicity and a temporal delay of the effector function^28^. It is possible that like what has been proposed for TcdB, the catalytic fragments of AT and PT are still translocated into the cytosol, however they remain tethered to the endosome due to the non-cleaving loop joining them to the B fragment. In this scenario, the C domain would have far less freedom of movement within the cell but may nonetheless engage its target (eEF-2). From an evolutionary standpoint, it is unlikely that the formation of the disulfide linkage could occur spontaneously with the furin cleavage motif. The most likely scenario is that the disulfide bond preceded the protease cleavage site, as the inverse order would result in the C domain being released and trapped in the endosome. AT and PT may thus reflect an early diverging lineage of DT-like toxins that lack some of the key characteristics that emerged in the DT lineage.

Understanding how the T-domain of DT evolved the ability to deliver DT_C_ across the endosomal membrane remains an open question for DT along with most other similarly translocating toxins. Uncovering the determinants of translocation has implications for understanding how proteins interact with and cross membranes to cause disease as well as for rational development of intracellular delivery vectors. We found that AT and PT possess marginally functional translocases with a lower pH threshold for unfolding and pore formation in vitro. Sensing of the endosomal pH by titratable residues within the T-domains has long been considered the first step in the process of translocation. Of the three groups of titratable amino acids, only histidine (pKa 6) and the acidic residues, aspartic and glutamic acid (pKa ~4), are expected to be protonated during endosomal acidification. All other DT-like proteins identified to date contain at least six His residues in their respective T domains^6^, presumably serving equivalent roles. The mechanistic role of these six His residues have been studied extensively^16, 18, 19, 29^. The complete lack of His residues in AT_T_ and an altered arrangement of His residues in PT_T_ relative to DT_T_ suggests that initiating the process of unfolding and pore-formation at higher pH’s may lead to more efficient endosomal escape and subsequent translocation of DT in human cells (Fig. 6e). It should be noted, given the lower pH threshold of unfolding and insertion for AT and PT, the contributions of the acidic residues within their T-domains are likely to be responsible for initiating the pH-induced unfolding. The lack of His residues in AT_T_ and low number in PT_T_ thus represent a shared feature that may be due to one of two evolutionary scenarios: either the shared precursor of all DT-like proteins contained few or no His residues in its T domain, and His residues were acquired in the α-clade, optimizing the pH transition to escape from the early endosome prior to lysosomal fusion; or DT-like proteins of the β-clade have lost His residues present in the shared ancestral toxin. In other words, these characteristics of AT and PT may be representative of a more ancient lineage of the DT family tree that are simply less efficient translocases, or their preferred hosts occupy a particular niche or possess a particular biology which would make a lower pH transition optimal for endosomal escape.

While other toxins may possess distant homology with regions of DT (i.e., conserved ADPRT activity), AT and PT represent the first true homologs of DT to be characterized structurally as they are the first to possess a complete DT-like three-domain architecture. This is consistent with our earlier sequence-based analysis, which placed AT and PT among the closest phylogenetic neighbors of DT outside of *Corynebacterium*. Despite widely divergent sequences, both AT and PT display remarkable structural conservation of overall domain architecture as well as within individual domains. We demonstrate that while DT shares several features with distantly related DT-like toxins targeting unknown (non-human) hosts, it also possesses features that are absent from these relatives through which we can potentially trace the emergence of human-specificity, and therefore the origins of the disease diphtheria. More broadly, the field of bacterial toxins has historically assumed a human-centric approach, as toxins have been identified primarily from clinical isolates. With the abundance of easily accessible and ever-expanding genomic databases, a new paradigm for toxin discovery has been enabled.

Recently, DeepMind made available the code for their AlphaFold network, which has been demonstrated to produce highly accurate predictions for a vast number of protein structures^30^. At the time of this writing (November 2021) DeepMind, in collaboration with EMBL-EBI, have made available structural predictions of >365,000 proteins from 21 model organisms including the entire human proteome. To see how accurately the AlphaFold network predicted the structures of of AT and PT, we submitted their sequences to ColabFold, a public Google Colab Notebook^31^. Interestingly, of the three domains, the AlphaFold-predicted R domain structures were closest to the AT and PT structures solved here among all of the domains (Supplemental Figs. 5 and 6). The structures of the C domains were also well predicted in terms of overall fold (Supplemental Figs. 5 and 6). By contrast, the T domains, while predicted to be entirely helical, do not assemble in the same manner as in the crystal structures. Rather than forming a tight, multi-layered bundle of helices wrapped around each other, the helices are predicted to adopt an orientation closer to parallel. Additionally, the interdomain contacts are poorly predicted, such that the overall fold of the AlphaFold prediction is quite different from the crystal structures. This result may be expected given that it is known that the accuracy of the prediction is affected by the number of sequences available to construct the multiple sequence alignment, and at the time of this writing there are <20 unique DT-like sequences available in the NCBI database. The inability to accurately predict both the general tertiary structure and specifically the tertiary structure of the T domain could also speak to the structural plasticity inherent in these toxins, which presumably adopt distinct structures upon formation of the molten globule state at low pH and subsequent membrane insertion.

Decades of studies on DT and other toxins with intracellular targets that mediate their own entry into cells, such as botulinum neurotoxin, tetanus toxin and anthrax toxin, have helped us to understand in detail how these proteins intoxicate at the molecular and cellular level to ultimately cause disease. By contrast, the origins of these toxins and their adaptation to the human host are fundamental questions that have not been readily addressable owing to the paucity of distant homologs adapted to different hosts to compare to. In this work, through the structural and functional characterization of two recently identified distant homologs of DT not adapted to human hosts, we uncovered insights into the evolution of DT and determinants of specificity to humans. We found that unlike other bacterial toxin families that have slight variations in enzyme function and intracellular targets, the DT-like family of proteins all conserved their catalytic domain function and target. We also found that much like how viruses jump from one host species to another through a series of stepwise evolutionary events that enable virus entry and replication, DT, and perhaps bacterial toxin specificity, for humans appears to derive from adaptations to host factors implicated in the entry process (i.e., receptor binding and subsequent internalization and enzyme release). Whereas receptor-switching through alterations in the receptor-binding surface may be sufficient to change tissue tropism within a given species (as is the case with the Large Clostridial Toxin family [32]), our work here seems to suggest that adaptation to multiple host-specific factors and processes implicated in toxin uptake (i.e., receptors, endosomal pH, activating proteases, etc.) are required to produce maximal toxicity toward a given host. Finally, this work demonstrates the importance of structural plasticity in the evolution of not just DT and DT-family proteins, but perhaps all bacterial toxins.

## Methods

### Chimera generation and protein purification

*E. coli* codon optimized gBlocks® gene fragments for full-length DT, AT and PT were purchased from Integrated DNA Technologies (IDT). Primary amino sequences were obtained for AT and PT from NCBI database entries WP_095582082.1 and WP_073156187.1, respectively. Individual domains were amplified by PCR, and domain-swapped chimeras were generated with the NEBuilder® HiFi DNA Assembly Cloning Kit (New England BioLabs). All toxins and chimeras were expressed and purified with cleavable N-terminal 6His-SUMO fusions (ThermoFisher), grown to an OD_600_ of 0.8 in LB media, and induced for 18 h at 18 °C using 0.1 mM IPTG. Cell pellets were pelleted by centrifugation and then re-suspended in lysis buffer (20 mM Tris pH 8.0, 500 mM NaCl, 1% protease inhibitor cocktail (Sigma), 1 mg/mL lysozyme, 0.01% benzonase (Sigma)) and lysed by 3-passes through an Emulsiflex C3 (Avestin) at 15,000 psi. Cell lysates were clarified by centrifugation (20 minutes at 18,000 × *g*) and bound to a 5 mL HisTrap™ FF Crude column (GE Healthcare) and eluted with 50–75 mM imidazole. Eluted protein was diluted to ~150 mM NaCl and incubated overnight at 4 °C with SUMO protease to cleave the affinity tag. Cleaved protein was separated from SUMO, SUMO protease and uncleaved protein with Ni-NTA resin, concentrated by centrifugation and exchanged into 20 mM Tris pH 7.5, 150 mM NaCl by dialysis.

### Protein synthesis inhibition

Vero cells (ATCC) were transduced with a lentivirus expressing NanoLuc® luciferase (Vero-NLucP cells) as described previously^9^. Vero-NLucP cells were plated (5000 cells/well) in a white clear-bottom 96-well plate and treated with toxin the following day. Following overnight incubation (20 h), luminescence signal was developed using the NanoGlo® Luciferase Assay kit (Promega) and read with a Spectramax M5e plate reader (Molecular Devices). Relative luminescence units (RLU) were normalized relative to untreated cells and data was analyzed with GraphPad Prism 7.0. For protein synthesis inhibition assays in HEK293T cells, cells were transiently transfected with the pNL3.2CMV (Promega) rather than by lentiviral transduction. Cells were plated in a 6-well plate at 1 × 10^6^ cells/well and transfected the following day with 3.0 µg of plasmid DNA, in a 3:1 ratio of FuGENE^®^HD (Promega) transfection reagent to DNA. After 24 h, cells were re-plated at 5 × 10^3^ cells/well, in a white clear-bottom 96-well plate and treated the following day with toxin. After overnight incubation (20 h), luminescence signal was developed using the NanoGlo® Luciferase Assay kit (Promega) similar to above. Each construct was tested 3 times with 2 technical replicates (at a minimum). Wildtype and DPH4^-/-^ HEK293T cells were a gift from Dr. Mikko Taipale at the University of Toronto.

### Cell viability

Cells (Vero-NLucP, HEK293T, ARN8) were plated in a black clear-bottom 96-well plate (5×10^3^ cells/well) and treated with toxin the following day. After 48 h, cells were treated with PrestoBlue™ reagent (Thermo Fisher Scientific), incubated for 2 h, and fluorescence was read (555 nm excitation and 585 nm emission) with a Spectramax M5 plate reader (Molecular Devices). Fluorescence measurements were normalized relative to untreated cells and data was analyzed with GraphPad Prism 7.0.

### Crystallization

All crystals were grown by hanging-drop vapor diffusion at 20 °C. For AT, 1 µL of selenomethionine derivatized AT (11.8 mg/mL) was mixed with 1 µL of mother liquor (10 mM Tris-HCl pH 7.0, 200 mM calcium acetate hydrate, 20% PEG3000) and dehydrated over 200 µL of mother liquor. Diffraction quality AT crystals were obtained following successive rounds of micro-seeding and flash frozen in liquid nitrogen without any additional cryoprotectant. PT crystals were grown from drops containing 1 µL PT (15.8 mg/mL) and 1 µL mother liquor (100 mM potassium iodide, 22% PEG3350) dehydrated over 200 µL of mother liquor. PT crystals used for data collection were grown following successive rounds of micro-seeding in drops dehydrated over 550 mM ammonium sulfate, and flash frozen in liquid nitrogen without any additional cryoprotectant. Datasets were collected remotely at a wavelength of 0.979 Å for AT crystals and 2.0 Å for PT crystals on the AMX beamline at NSLSII (Brookhaven National Labs).

### Structure solution

All diffraction data was processed with XDS using the AutoPROC package^32^. Structure solution of AT by SAD phasing was carried using the CRANK2 pipeline^33^ in CCP4^34^. The initial solution yielded 39 heavy atom sites (selenium) with an FOM of 56.8, producing a partial model with an *R*_factor_ of 43.37% (Buccaneer). A single monomer from the partial model was used to perform molecular replacement using the PHENIX software package^35^, which located 4 copies of AT in the asymmetric unit, which were then re-built with PHENIX-AutoBuild. SAD phasing of PT was performed with PHENIX-AutoSol, yielding 18 heavy atom sites (iodine) with an FOM of 35.2 and a partial model (monomer) with an *R*_factor_ of 48.6%. Model building and refinement was carried out through multiple iterations of manual building in Coot^36^ and PHENIX-Refine until *R* and *R*_free_ values converged and geometry statistics reached suitable ranges.

### Sequence alignments

Sequence identity and similarity between DT, AT, and PT were calculated from a multiple sequence alignment generated with the T-coffee server using the M-coffee algorithm (http://tcoffee.crg.cat/)^37^.

### Phylogenetic tree construction

In order to compare DTs and their homologs phylogenetically, a representative set of DTs from *Corynebacterium* spp. were combined with DT homologs from *Streptomyces albireticuli* (protein accession number WP_095582082.1) and *Seinonella peptonophila* (WP_073156187.1). Multiple sequence alignments of full-length proteins were created using MAFFT (version 7.487; the high-accuracy L-INS-i algorithm) [39], ClustalO (version 1.2.4)^38^, MUSCLE (version 3.8.1551)^39^, and PRANK (version v.170427; with the -F flag to maintain insertions over alignment iterations)^40^. Maximum likelihood phylogenetic trees were generated for each of these alignments with RAxML (version 8.2.12)^41^. Each RAxML tree used rapid bootstrapping followed by a slow maximum likelihood search, the autoMRE bootstrapping algorithm, automatic amino acid substitution matrix selection, and the GAMMA model of rate heterogeneity. The tree that yielded the highest likelihood was selected as the representative topology. The topology of the RAxML tree was further supported through Bayesian phylogenetic inference with MrBayes (version 3.2.7)^42^. MrBayes was run with the GTR model and gamma-distributed rate heterogeneity, the “mixed” amino acid substitution matrix (allowing the program to sample from the available matrices and select the best one), 3 runs with random starting trees and a maximum chain length of 1,000,000 generations, automatic simulation stopping via average standard deviation of split frequencies, and a 25% burn-in fraction. The posterior support of clades of the resulting consensus tree are depicted in Fig. 1. All scripts needed to reproduce the bioinformatic analysis can be found at https://github.com/mjmansfi/Sugiman-Marangos_Gill_etal_2021.

### Vero lysate processing assay

Vero-NLucP cells (3 × 10^6^) were resuspended in 500 µl lysis buffer (0.05% Triton X100 in PBS). In all, 2.5 µg of toxin was incubated with 0.5 µL of cell lysate overnight at 37 °C in a total volume of 5 µL PBS with 2 mM CaCl_2_. The reaction was stopped by adding 5 µL of 2× SDS-PAGE loading dye. Samples were boiled for 3 min and separated on a 4–20% precast polyacrylamide gel (Biorad).

### TNS fluorescence assay

2-(p-toluidino) naphthalene-6-sulfonic acid (TNS) assays were performed as described previously^43^. Briefly, proteins (20 nM final concentration) were incubated in 150 mM citrate phosphate buffers ranging from pH 3.0 to 7.5 with 150 µM 2-(*p*-Toluidinyl) naphthalene-6-sulfonic acid, sodium salt (Invitrogen). Following 25-min incubation at 37 °C, fluorescence was read (366 nm excitation and 400–500 nm emission scan) with a Spectramax M5 plate reader (Molecular Devices).

### DSF assay

DSF was performed as described previously^44^. Proteins were diluted to a final concentration of 0.1 mg/mL in 150 mM citrate phosphate buffers ranging from pH 3.0 to 7.5 containing 5× SYPRO Orange (Invitrogen). SYPRO Orange fluorescence was monitored over a 25–80 °C temperature gradient (30 s increments) using a BioRad CFX96 qRT-PCR thermocycler.BioRad CFX Manager 3.1 was used to integrate fluorescence curves and calculate melting temperatures.

### Liposomal dye release assay

Unilamellar liposomes (DOPC, 0.8% DGS-NTA, Avanti Polar Lipids) were prepared as previously described^5^. Specifically, 1,2-dioleoyl-sn-glycero-3-phosphocholine (DOPC) (Avanti Polar Lipids) was combined with 0.8% 1,2-dioleoyl-sn-glycero-3-[(*N*-(5-amino-1-carboxypentyl)iminodiacetic acid)succinyl] (nickel salt) (DGS-NTA[Ni]) (Avanti Polar Lipids), dried with N_2_ and incubated for 1 h in a vacuum desiccator. Lipids were resuspended in 20 mM Tris pH 8, 35 mM 8-Hydroxypyrene-1,3,6-trisulfonic acid (HPTS), 50 mM p-xylene-bis-pyridinium bromide (DPX) (Thermo Fischer) and subject to 10× freeze–thaw cycles in dry ice and 42 °C water bath, and 10× extrusions using a 200 µm filter. The liposomes were then purified by gel filtration using a Hi Prep 16/60 Sephacryl S-300 HR column (GE Healthcare) in 150 mM NaCl, 20 mM Tris pH 8 buffer. Proteins were added in a ratio of 1:10,000 with liposomes, with a final liposome concentration of ~400 µM, in 150 mM citrate phosphate buffer ranging from pH 4.0 to 7.5, in 0.5 pH intervals. Assays were done in 96-well opaque plates (Corning), and fluorescence was monitored over a 20-min interval, with readings being taken every 30 s (excitation 403 nm, emission 510 nm). Data were normalized to the percentage of total HPTS fluorescence, by adding 0.3% Triton X-100.

### Generation of bulk ARN8 HBEGF_KO_ cell line

crRNA targeting the HBEGF (5′-ACGGACCAGCTGCTACCCCT) was designed using the Integrated DNA Technologies (www.idtdna.com) design tool. The gRNA:Cas9 ribonucleoprotein complex was assembled according to the manufacturer’s protocol (Integrated DNA Technologies) and reverse transfected using Lipofectamine RNAiMAX (Thermo Fisher Scientific) into ARN8 cells (3 × 10^5^ cells in a 6-well plate). Following 48 h of incubation, 10 nM of WT DT was added to the plates to select for HBEGF knockout cells.

### hHBEGF overexpression in ARN8 HBEGF_KO_ cells

The gene for human HBEGF was synthesized and cloned into the MCS of PiggyBac vector PB-CMV-MCS-EF1α-Puro (System Biosciences) by restriction digest with EcoRI and BamHI (pCMV-hHBEGF). ARN8 HBEGF_KO_ cells were transfected at 80% confluence in a 6-well plate with pCMV-hHBEGF (2 µg) and PB Transposase vector (0.5 µg) (System Biosciences) using FuGENE® HD (Promega) as per the manufacturer’s protocol. Selection was performed with puromycin (6 µg/mL) for 1 week beginning 48 hours after transfection.

### Statistics and reproducibility

All statistical calculations (mean and standard deviation) were calculated by GraphPad Prism 7.0. All biophysical, protein synthesis and cell viability experiments were repeated a minimum of 3 times with 2 or more technical replicates performed in each experiment. Data presented in figures constitute representative data. Data from all biological replicates are available in the Supplemental Data file.

### Reporting summary

Further information on research design is available in the Nature Research Reporting Summary linked to this article.

## Supplementary information

Supplemental material

Description of Additional Supplementary Files

Supplemental Data

Reporting Summary

## Data Availability

All structural data generated during the current study for AT and PT can be found in the Protein Data Bank (https://www.rcsb.org) under the accession numbers 7RIS and 7RB4. All other data generated or analyzed during this study are available in Supplemental Fig. 7 and within the Supplemental Data.

## Materials availability

Unique DT chimeric constructs generated in this current study are available from the corresponding authors on reasonable request.
