# Supplementary material for: Structures of distant diphtheria toxin homologs reveal functional determinants of an evolutionarily conserved toxin scaffold
Source: Commun Biol. 2022 Apr 19;5:375. doi: 10.1038/s42003-022-03333-9 (PMC9018708; doi:10.1038/s42003-022-03333-9)
Supplement: Supplementary file 1 — Supplemental material [file 42003_2022_3333_MOESM1_ESM.pdf]

Supplemental Material

Structures of distant diphtheria toxin homologs reveal functional determinants of an evolutionarily conserved toxin scaffold

Seiji N. Sugiman-Marangos[†1], Shivneet K. Gill[†1,2], Michael J. Mansfield[3], Kathleen E. Orrell[1,2], Andrew C. Doxey[4], and Roman A. Melnyk[1,2,*]

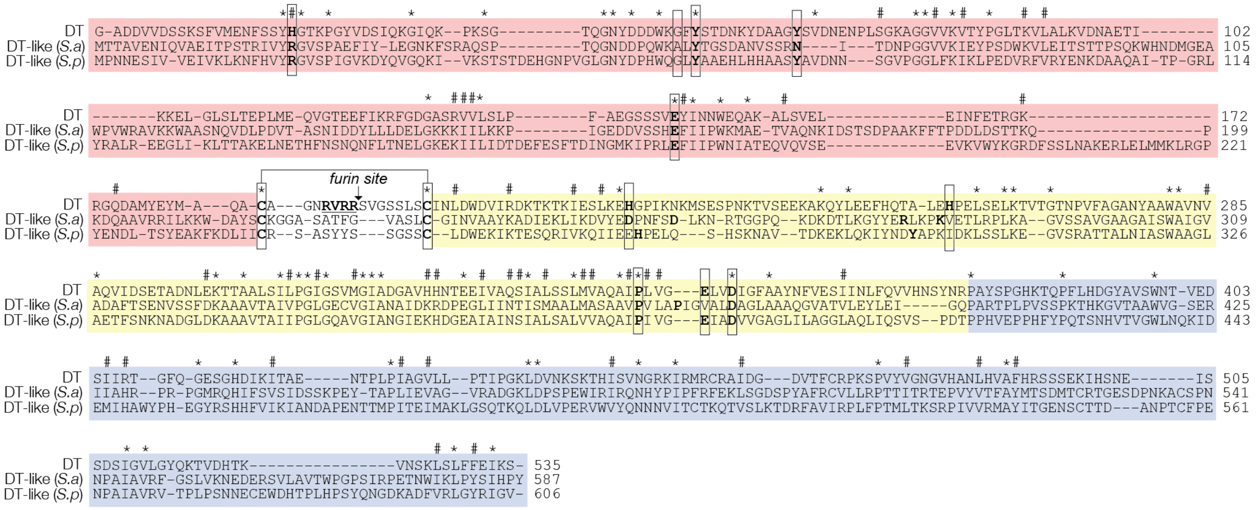

Supplemental Figure 1. Sequence alignment.

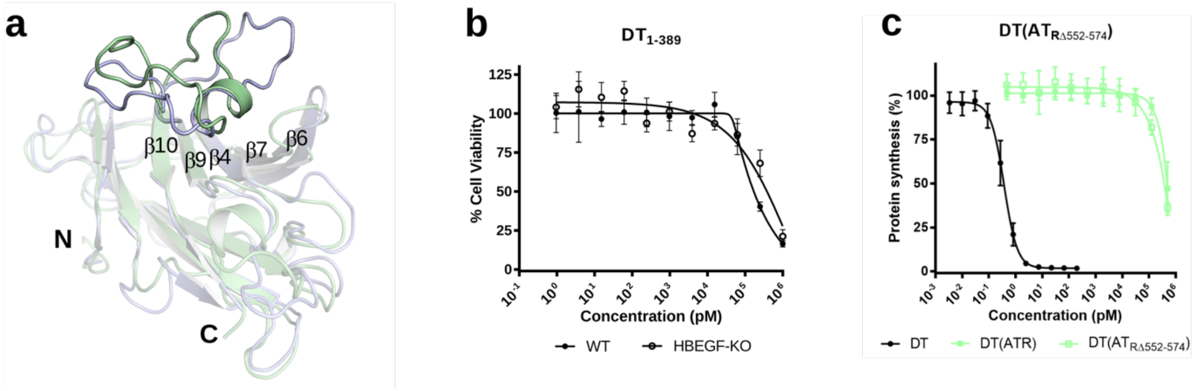

Supplemental Figure 2. R domain. (**a**) Structural differences in the lid structure present in AT and PT. In PT_R the 'lid' is more splayed, and the apex of the loop does not possess as much helical character while in AT_R, it adopts a full helical-turn. Sequence conservation between the two proteins in this region is low (11% identical, 22% similar) relative to the full domain (28% identical, 42% similar). (**b**) Dose titration of $DT_{1\text{-}389}$ on WT and $HBEGF_{KO}$ cells (mean ± SD, n=3). Sensitivity to $DT_{1\text{-}389}$ was unaffected by HBEGF expression (EC_{50}'s: WT-207 nM, $HBEGF_{KO}$-498 nM). (**c**) The 'lid' sequence in AT_R (residues 552-574) was replaced with two Gly residues in the DT(AT_R) chimeric construct. Dose titration of DT, DT(AT_R), and DT($AT_{R\Delta552\text{-}574}$) on Vero cells (mean ± SD, n=3).

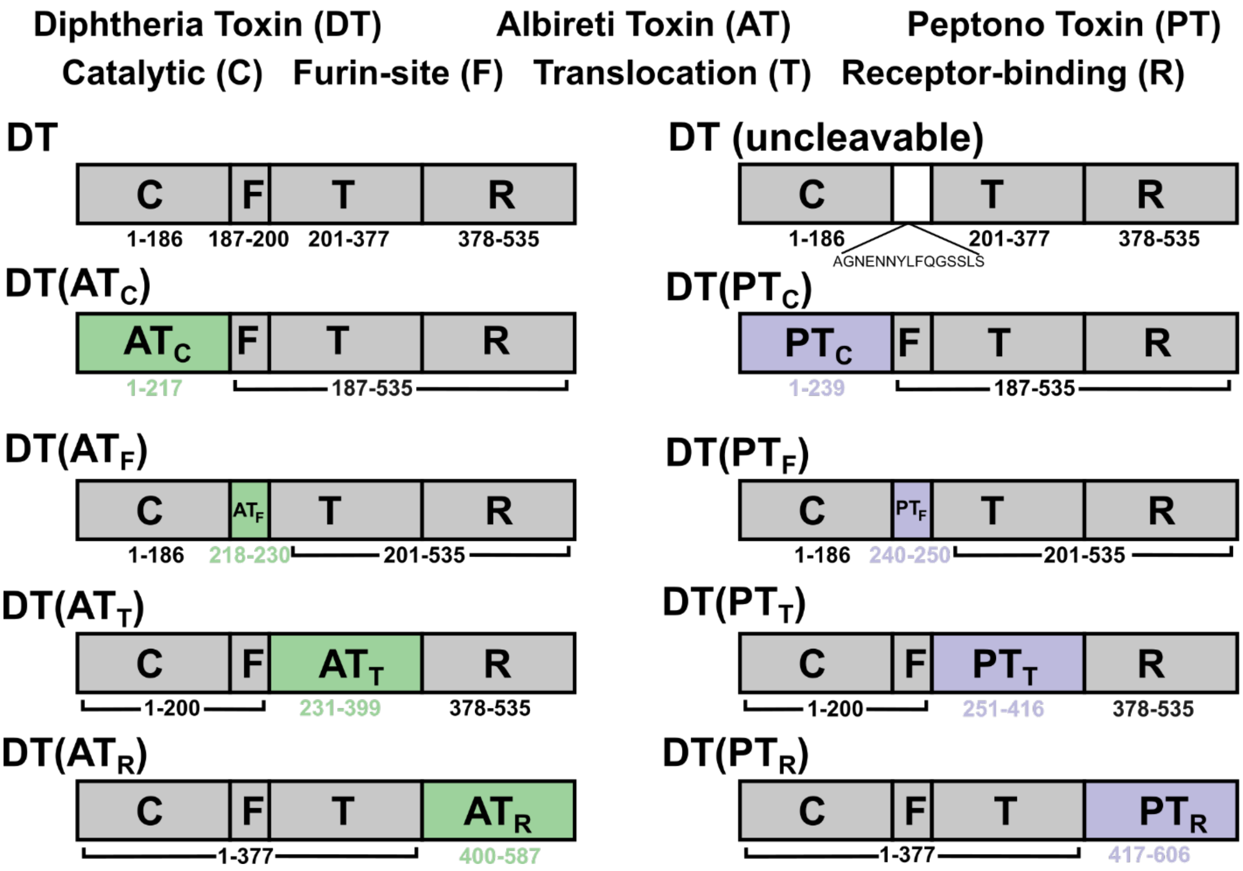

Supplemental Figure 3. DT chimeras. Architecture and residue numbering of DT domain swapped chimeras.

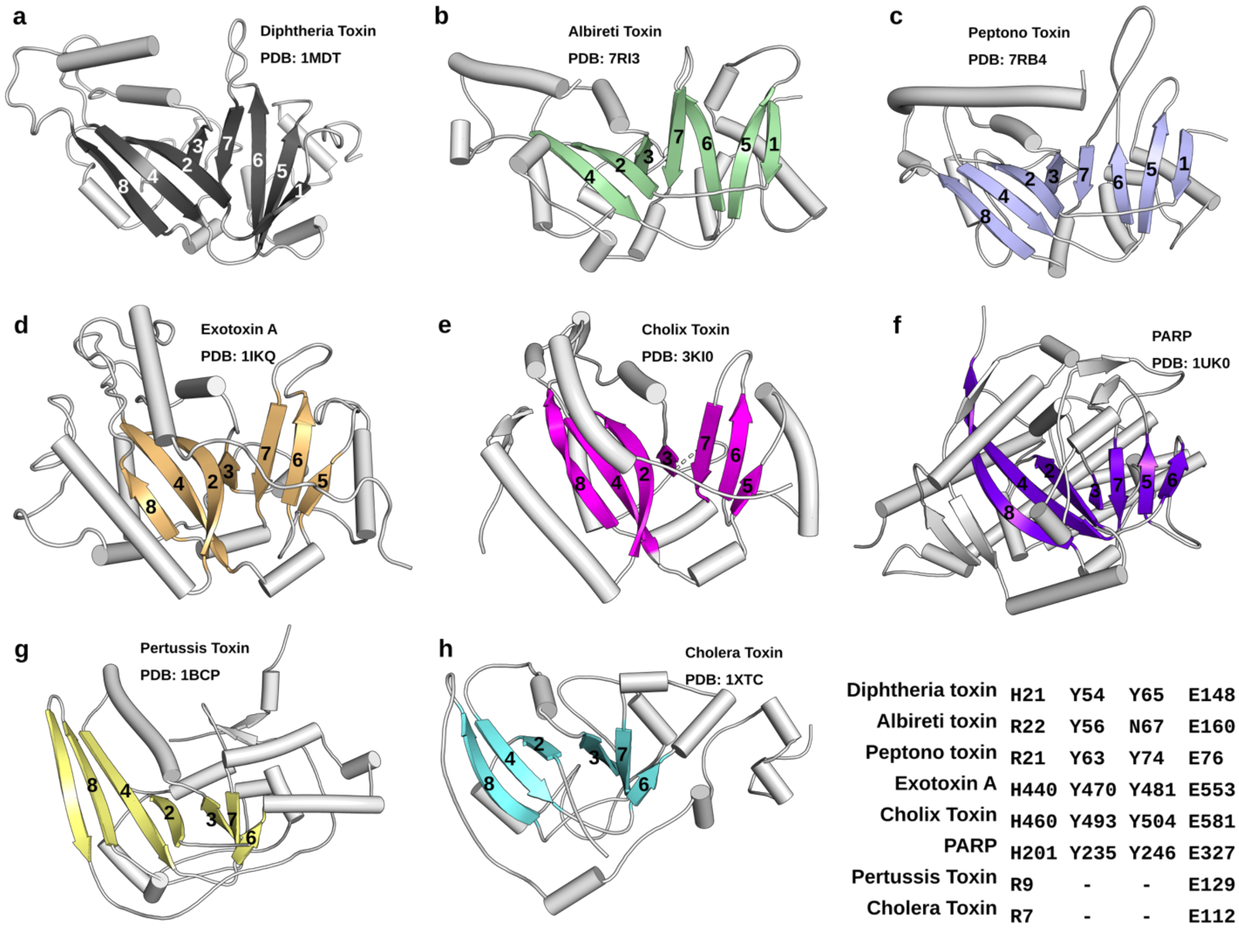

Diphtheria toxin	H21	Y54	Y65	E148
Albireti toxin	R22	Y56	N67	E160
Peptono toxin	R21	Y63	Y74	E76
Exotoxin A	H440	Y470	Y481	E553
Cholix Toxin	H460	Y493	Y504	E581
PARP	H201	Y235	Y246	E327
Pertussis Toxin	R9	-	-	E129
Cholera Toxin	R7	-	-	E112

Supplemental Figure 4. ADP ribosylating enzyme folds. Conservation of the split β-sheet amongst ART family catalytic domains.

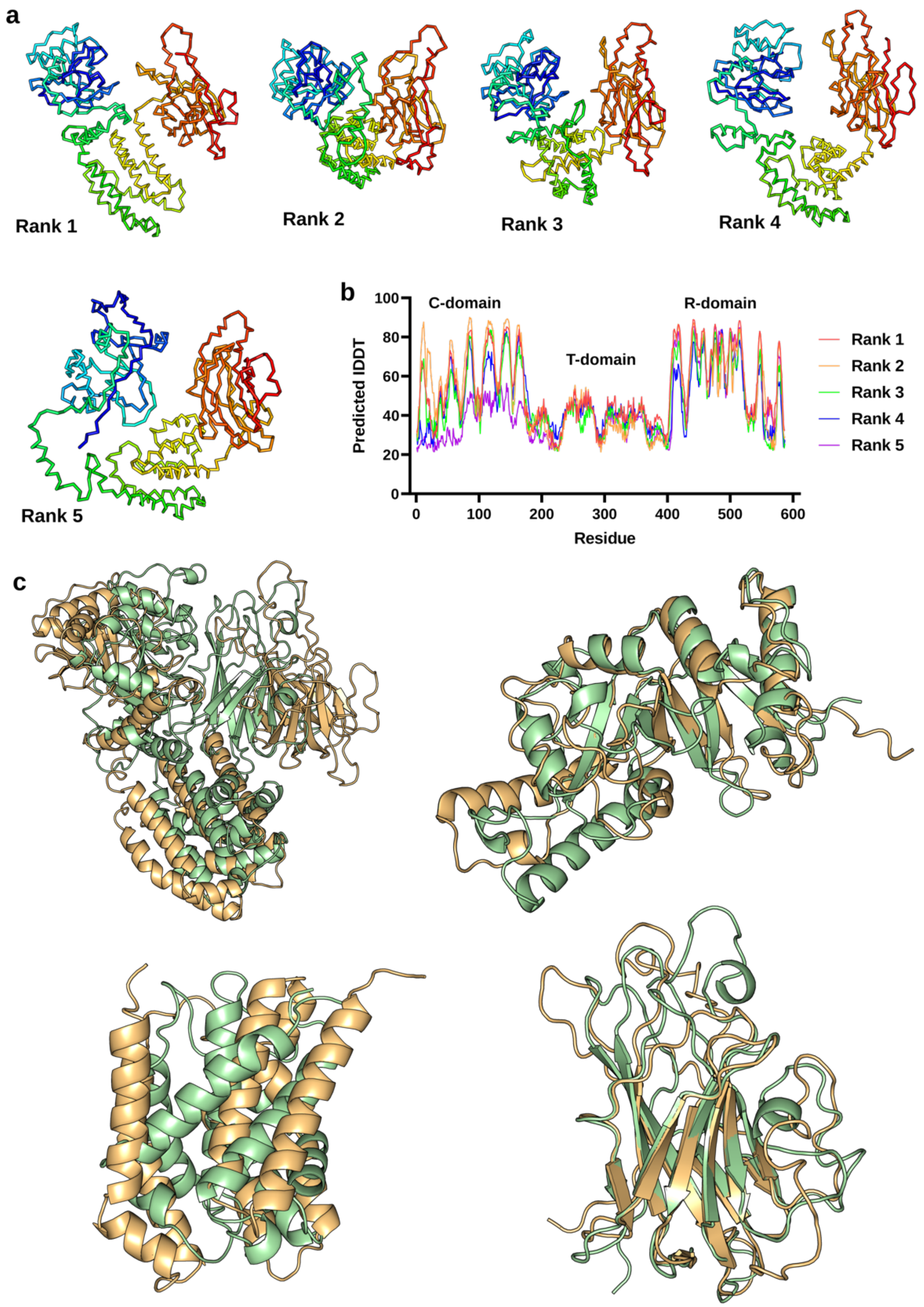

Supplemental Figure 5. AT Prediction – (**a**) 5 models of AT generated by the AlphaFold network using ColabFold coloured N→C (blue to red). (**b**) Predicted lIDT scores by residue number for models ranked 1 to 5. (**c**) Superpositions of top ranked AlphaFold prediction with the crystal structure of AT: holotoxin (top-left), C-domain (top-right), T-domain (bottom-left), R-domain (bottom-right).

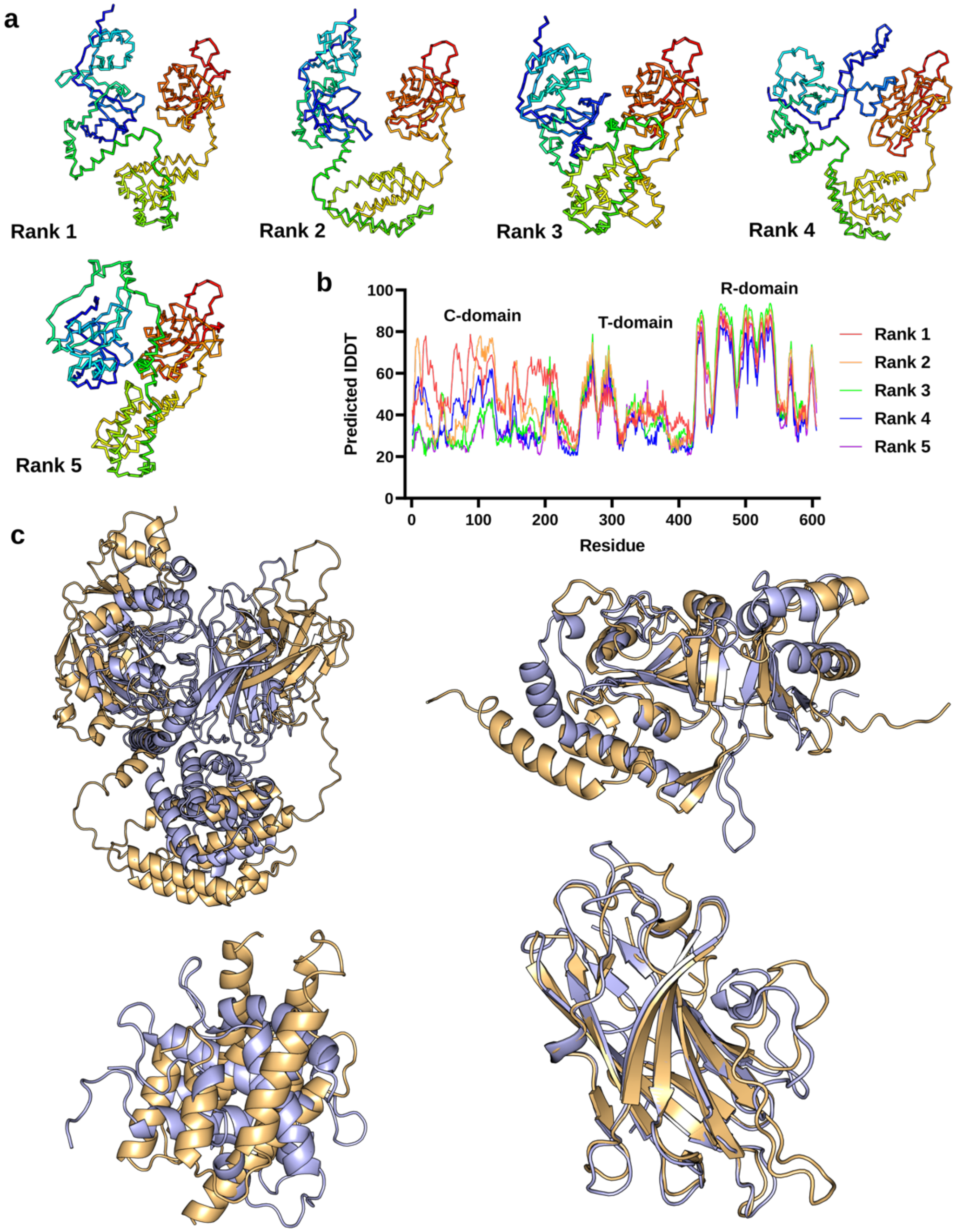

Supplemental Figure 6. PT Prediction – (**a**) 5 models of PT generated by the AlphaFold network using ColabFold coloured N→C (blue to red). (**b**) Predicted lDDT scores by residue number for models ranked 1 to 5. (**c**) Superpositions of top ranked AlphaFold prediction with the crystal structure of PT: holotoxin (top-left), C-domain (top-right), T-domain (bottom-left), R-domain (bottom-right).

a

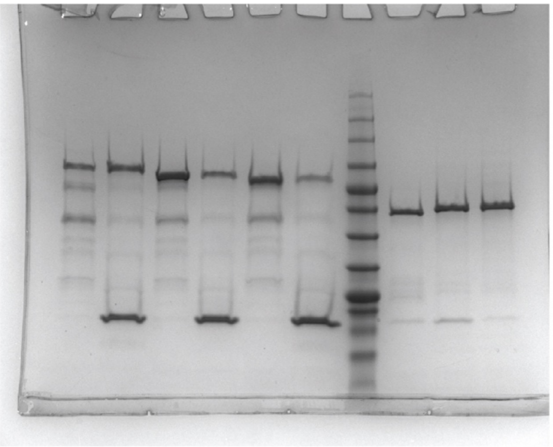

b

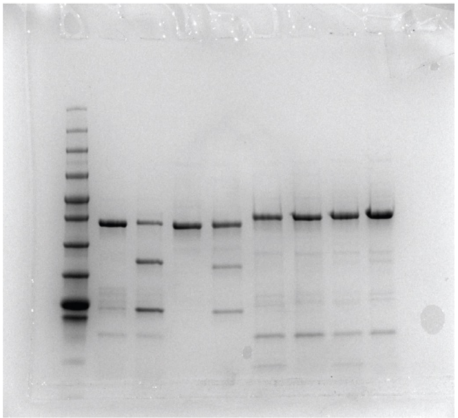

Supplemental Figure 7. Uncropped gels used in this work. (**a**) uncropped gel from which gel presented in Figure 1f was derived. Only Lanes 7-10 were used in figure. Lane 7 corresponds to molecular weight markers. Lane 8 is purified DT; Lane 9 is purified AT; Lane 10 is purified PT (**b**) uncropped gel from which gel presented in Figure 5c was derived. Only Lanes 1-3 and 6-9 were used were used in figure. Lane 1 corresponds to molecular weight markers. Lane 2 is DT – lysate; Lane 3 is DT + lysate; Lane 6 is AT -lysate; Lane 7 is AT + lysate; Lane 8 is PT – lysate; Lane 9 is PT + Lysate.
