# Supplementary material for: Structures of distant diphtheria toxin homologs reveal functional determinants of an evolutionarily conserved toxin scaffold
Source: Commun Biol. 2022 Apr 19;5:375. doi: 10.1038/s42003-022-03333-9 (PMC9018708; doi:10.1038/s42003-022-03333-9)
Supplement: Supplementary file 2 — Description of Additional Supplementary Files [file 42003_2022_3333_MOESM2_ESM.pdf]

Description of Additional Supplementary Files

File name: Supplemental Data
Description: Data from all biological replicates.
